# The Effects of Gender Differences in Patients with Depression on Their Emotional Working Memory and Emotional Experience

**DOI:** 10.1155/2015/807343

**Published:** 2015-10-22

**Authors:** Mi Li, Shengfu Lu, Gang Wang, Ning Zhong

**Affiliations:** ^1^International WIC Institute, Beijing University of Technology, Beijing 100124, China; ^2^Beijing International Collaboration Base on Brain Informatics and Wisdom Services, Beijing 100124, China; ^3^Beijing Key Laboratory of MRI and Brain Informatics, Beijing 100053, China; ^4^Mood Disorders Center & China Clinical Research Center for Mental Disorders, Beijing Anding Hospital, Capital Medical University, Beijing 100088, China; ^5^Center of Depression, Beijing Institute for Brain Disorders, Beijing 100088, China; ^6^The Department of Life Science and Informatics, Maebashi Institute of Technology, Maebashi 371-0816, Japan

## Abstract

A large amount of research has been conducted on the effects of sex hormones on gender differences in patients with depression, yet research on cognitive differences between male and female patients with depression is insufficient. This study uses emotion pictures to investigate the differences of the emotional working memory ability and emotional experience in male and female patients with depression. Despite identifying that the working memory of patients with depression is impaired, our study found no significant gender differences in emotional working memory. Moreover, the research results revealed that memory effects of mood congruence are produced in both men and women, which may explain why the depression state can be maintained. Furthermore, female patients have more emotional experiences than male patients, which is particularly significant in terms of negative emotional experiences. This result provides cognitive evidence to explain why women suffer from longer terms of depression, are more susceptible to relapse, and can more easily suffer from major depressive disorder in the future.

## 1. Introduction

The causes of depression are very complex, affected by many factors including the biological, social, and psychological factors. Considering the differences of biological construction, social roles, and psychological structure between men and women, women are more likely to experience mood disturbances during times of hormonal flux. The etiologic model of depression with onset in the menopause transition proposed by Gordon et al. [1] is very helpful for understanding the female depressive mood. This model shows that in the context of the menopause transition, characterized by fluctuations in ALLO that are consequent to estradiol and progesterone fluctuations, an inability of the GABAA receptor to demonstrate the plasticity necessary to maintain GABA-ergic homeostatic control might exacerbate the response of the HPA axis to stress. Combined with an increased vulnerability to major depressive disorder due to personality or genetic factors and/or stressful life events proximate to the menopause transition, the endocrine profile of the menopause transition sets the stage for depressive symptoms. As a result, the possibility of women suffering from an emotional illness is far greater than that of men [2]. Studies have demonstrated that, compared to men, women with depression have a higher point prevalence of depression, an earlier onset of depression [3], and a higher risk of severe depressive disorder in the future [4, 5]. In addition, females have longer lasting depression and are more likely to experience relapse [6]. There have been many studies on the biological factors that influence gender differences in patients with depression. For example, no significant difference in the risk of depression has been found between postmenopausal women and men [7, 8], indicating that the secretion levels of androgen and estrogen affect a person's psychoactive state. During stages of hormonal fluctuations and instability, such as adolescence and postpartum, women have relatively strong mood fluctuations and are prone to experiencing anxiety and depression [9–11]. In the context of the menopause transition, neurosteroids (including estradiol, progesterone, and GABA-ergic) fluctuation/dysregulation might increase the response of the HPA axis to stress. Combined with an increased pressure due to personality of the female or genetic factors and/or stressful life events, these physiological and social factors interacting with each other might lead to the occurrence of depression [1]. Clinical studies on males show that the hormone testosterone provides protective benefits against anxiety and depression. A decline in male testosterone levels is accompanied by a significantly higher incidence rate of anxiety and depression [12, 13]. Studies have shown that androgen, depleting drugs used to treat male prostate cancer, will lead to decreased male testosterone, which may result in increased risk of anxiety or depression [14]. For males with gonadal function decline, testosterone-replacement therapy is used, which greatly improves the patients' mood, reduces their anxiety, and alleviates their symptoms of depression [13, 15]. Similarly, among older men and women, low testosterone levels and an increased incidence rate of major depressive disorder are significantly associated [16, 17]. Although the male hormone testosterone level is significantly higher than that of the female (the content of testosterone in males is ten times that in females), in fact, women are more sensitive to testosterone [18]. The antianxiety and antidepression effects of testosterone have been supported by some evidence. For example, female patients with major depressive disorder or anxiety show low levels of salivary testosterone [19]. The use of low doses of testosterone in female patients with severe refractory depression has significantly alleviated their levels of depression [20].

All of the above-mentioned research examined affective disorders from the biological characteristics of sex hormone differences between males and females. In fact, biological characteristics influence cognitive psychology through social activities, interpersonal relationships, and so forth, thus forming stable psychological structures and cognitive styles of the different genders. Numerous studies show that gender differences in cognitive ability are considered to be an indisputable fact. Women have universal advantages in the tasks of speech production [21] and face memorization compared to men [22]. Men display a better ability than women in performing visuospatial tasks [23–25], which has mainly been demonstrated through the large difference in mental rotation ability and the small difference in visual-spatial perception ability [26]. Studies suggest that because of the inhibition effects of estrogen on the right brain, the ability for women to perform visuospatial tasks decreases [27]. Such gender differences in cognitive function may be related to the distribution of different hormone receptors in the brain. According to research, testosterone receptors are found in the hypothalamus and hippocampus [28, 29], whereas estrogen receptors are found in wider brain areas, such as the cerebral cortex, amygdala, hippocampus, and thalamus [30], suggesting that estrogen has greater effects on women's cognitive ability.

However, evidence of the association between gender differences and cognition is far from clear, particularly regarding cognitive differences between genders in patients with depression. Intuitively speaking, female patients with depression are more vulnerable to emotional control and influence compared to males, but evidence is lacking in this area, particularly evidence in the aspect of cognition. With a focus on female and male patients with first untreated depression and depression of a lesser extent, this study investigates differences in emotional working memory and emotional experiences between the two genders, thus providing more experimental evidence for a cognitive function comparison between male and female patients.

## 2. Methods

All subjects provided signed informed consent and this study was approved by the Ethics Committee at Beijing Anding Hospital, Capital Medical University, China.

### 2.1. Subjects

Twenty-three patients with depression participated in this experiment, including 12 females and 11 males. The patients arrived at the Outpatient Department of the Beijing Anding Hospital of Capital Medical University for their first visit. The patients with depression were diagnosed according to the Diagnostic and Statistical Manual of Mental Disorders (DSM-IV). The grouping criteria of these patients were as follows: (1) aged 18–60 years, right-handed, and fit the DSM-IV diagnostic criteria for depression; (2) diagnosed as patients with obvious, mild, and moderate depression; able to continue normal life, work, and study; (3) HAMD < 24; the level of depression was decided using the Hamilton Depression Rating Scale (HAMD-17); (4) did not receive any treatment; did not take any antidepressant drugs; (5) no color blindness or other eye diseases; had normal vision or corrected vision and were able to complete the eye movement test.

### 2.2. Experiment Materials

The experiment used 60 pictures each of positive, neutral, and negative images. All of the images were from the International Affective Picture System (IAPS). The average merriness of the positive pictures was 7.31 ± 0.44, and the average arousal was 5.54 ± 0.44. The average merriness of the negative pictures was 2.79 ± 0.51, and the average arousal was 5.97 ± 0.44. The average merriness of the neutral pictures was 5.18 ± 0.17, and the average arousal was 3.23 ± 0.22. After picture processing via Picture Manager software, the size, grayscale, and resolution of all of the pictures were the same.

### 2.3. Experimental Paradigm and Procedures

There were three types of experimental tasks: pictures of positive emotions, pictures of negative emotions, and pictures of neutral emotions. To simultaneously examine mood and emotional working memory, each type of task consisted of four pictures of the same type (i.e., each positive picture task was composed of four positive pictures, each negative picture task was composed of four negative pictures, and each neutral picture task was composed of four neutral pictures). The four pictures of each task corresponded with four different positions (upper left, upper right, lower left, and lower right). The target stimulus picture type was the same as the prompt task type; the target stimuli were presented at the center of the screen. During the experiment, a Tobii T120 Eye Tracker was used to simultaneously acquire and record the subjects' eye movement data, such as pupil diameter, while they were viewing the stimuli tasks.

The experimental procedure was as follows. First, a “+” sign appeared in the center of the screen for duration of 500 ms to remind the participants that the stimuli tasks would immediately appear. Then, the stimuli appeared for 10,000 ms to allow the subjects to remember all of the images in the clue. After the disappearance of the clue, there was a 5,000 ms memory retention time. Afterwards, a target image appeared in the center of the screen; the subjects stated whether the target image had appeared in the previous stimuli task. If their response was “yes,” they left-clicked; if their response was “no,” they right-clicked. An “*∗*” showed up upon the completion of judgment to indicate a break; the break time was 2,000 ms in between trials.

### 2.4. Statistical Analysis

A comparative analysis of the differences in age and years of education between the men and women was conducted using an independent sample *t*-test. In this study, the main factors were analyzed with the multivariate and multiple comparison correction method of generalized linear models. A pairwise comparative analysis of the same type of emotion between different gender groups was performed using an independent sample *t*-test. A pairwise comparative analysis of the same gender between groups of different emotional types was performed using a paired sample *t*-test. The statistical analyses of all of the data were conducted with SPSS 20.0 (SPSS, Inc., Chicago, IL) statistical analysis software.

## 3. Experimental Results and Analysis

### 3.1. Demographic Analysis

Eleven male patients and 12 female patients with depression participated in this experiment. Data from the demographic analysis are shown in Table 1. The difference in age distribution between male and female patients was not significant [*t*(21) = 0.126, *p* = 0.901] and the difference in the distribution of years of education between male and female patients was not significant [*t*(21) = 0.015, *p* = 0.989], but the difference in average HAMD scores (17 items) between male and female patients was significant [*t*(21) = 2.59, *p* = 0.01 < 0.05].

### 3.2. Comparison of the Accuracy of Working Memory between Male and Female Patients

**Figure 1:**
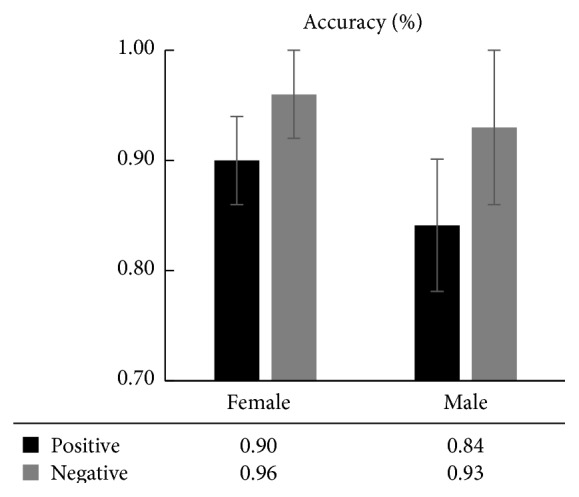
Comparison of the accuracy of the emotional working memory between men and women with depression.

The results of the average accuracy of working memory in the two genders are shown in Figure 1. We used the accuracy as the dependent variable and the gender factors and emotional factors as the independent variables. We then conducted 2 (gender: female, male) × 2 (emotion: positive, negative) two-way ANOVA. The results showed that gender main effects were not significant [*F*(1,21) = 1.410, *p* = 0.255, *η* ^2^ = 0.092], whereas the emotional main effects were significant [*F*(1,21) = 9.022, *p* = 0.008 < 0.01, *η* ^2^ = 0.361]; however, there was no interactive effect between gender and emotion [*F*(1,21) = 1.584, *p* = 0.229, *η* ^2^ = 0.102]. The results explain that the accuracy of the emotional working memory did not differ between male and female patients.

Additionally, we conducted a pairwise *t*-test on emotional factors. The results revealed that the accuracy of the women's negative emotional memory was significantly greater than that of their positive emotional memory [*F*(1,11) = 2.399, *p* = 0.035 < 0.05, *d*(effect size) = 0.899]; similar results were shown for men [*F*(1,10) = 3.000, *p* = 0.020 < 0.05, *d* = 0.966]. This indicates that both men and women with depression have memory effects of mood congruence; in other words, patients with depression remember more negative information that is consistent with their mood and less positive information [31, 32].

### 3.3. Comparison of the Reaction Time of Working Memory between Male and Female Patients

**Figure 2:**
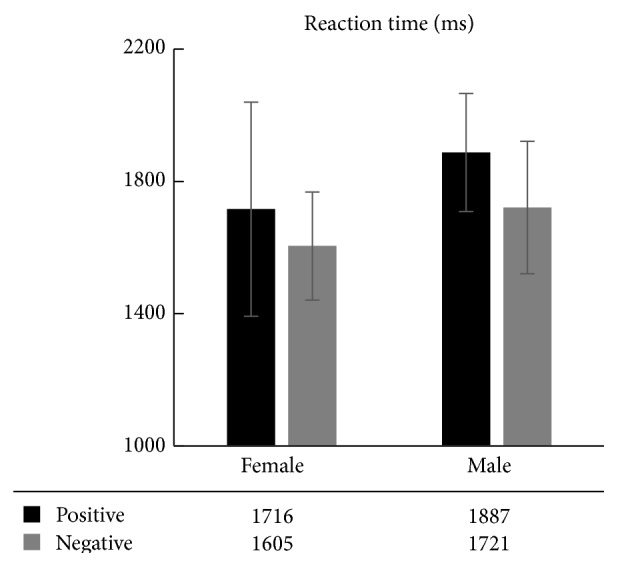
Comparison of the reaction time of emotional working memory between male and female patients with depression.

The results of the average reaction time of the working memory of men and women are shown in Figure 2. Using reaction time as the dependent variable and gender and emotion factors as the independent variables, we conducted 2 (gender: female, male) × 2 (emotion: positive and negative) two-way ANOVA. The results showed that the gender main effects were not significant [*F*(1,21) = 1.478, *p* = 0.244, *η* ^2^ = 0.096], the emotional main effects were not significant [*F*(1,21) = 1.228, *p* = 0.287, *η* ^2^ = 0.081], and the interactive effects between gender and emotion were not significant [*F*(1,21) = 6.339, *p* = 0.025 < 0.05, *η* ^2^ = 0.312]. The results indicated that no gender differences were demonstrated in the reaction time of emotional working memory between male and female patients.

Through the study on emotional working memory, we discovered that (1) both male and female patients with depression have memory effects of mood congruence, which is consistent with previous findings about the presence of the memory of mood congruence in patients with depression; and (2) although female patients had a stronger emotional working memory than male patients, no significant gender differences were produced.

### 3.4. Comparison of Changes in Pupil Diameter between Male and Female Patients

**Figure 3:**
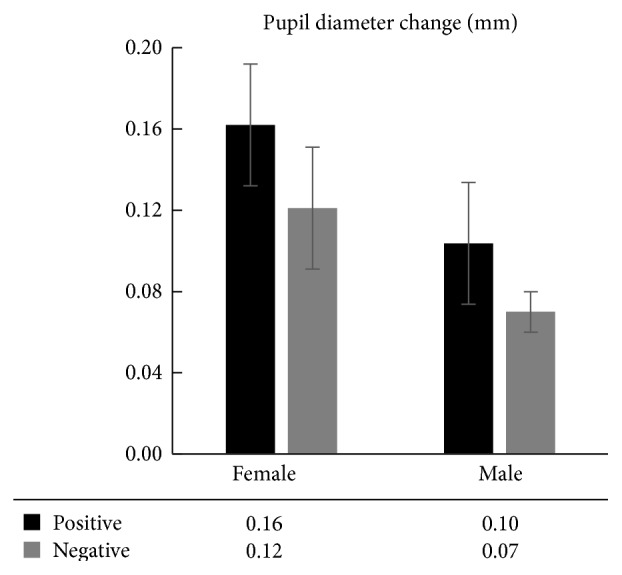
Comparison of pupil diameter changes between male and female patients with depression.

Using pupil diameter during a neutral emotion as the baseline, pupil diameter changes during individuals' positive and negative emotions were calculated. The results of the classified statistical analysis of the pupil diameter changes of different genders during different emotions are shown in Figure 3. Using pupil diameter changes as the dependent variable and gender and emotional factors as the independent variables, we conducted 2 (gender: female, male) × 2 (mood: positive, negative) two-way ANOVA. The results showed that the gender main effects were significant [*F*(1,21) = 4.911, *p* = 0.044 < 0.05, *η* ^2^ = 0.260], whereas the emotional main effects were not significant [*F*(1,21) = 2.216, *p* = 0.159, *η* ^2^ = 0.137] and the interactive effects between gender and emotion were not significant [*F*(1,21) = 0.472, *p* = 0.503, *η* ^2^ = 0.033].

The two-way ANOVA on pupil diameter changes showed that gender differences existed in pupil diameter changes. Therefore, further analysis was required regarding the significance of the difference in pupil diameter changes between men and women during different emotions. The results of the pairwise independent sample *t*-test showed that women had bigger pupil diameter changes than men during positive emotions, but the difference was not significant [*t*(21) = 1.409, *p* = 0.178, *d* = 0.709]. However, women had bigger pupil diameter changes than men during negative emotions, and the difference was significant [*t*(21) = 0.146, *p* = 0.048 < 0.05, *d* = 1.080]. The results suggest that gender differences exist in pupil diameter changes; this is represented in the discovery that women have significantly greater pupil diameter changes than men during negative emotions.

## 4. Discussion

This study examines the differences in the emotional working memory and emotional experiences between male and female patients with depression. To rule out the effects of age and years of education on the results, we matched the age and years of education of the men and women so that there was no significant difference in these two factors between genders.

Previous research results have shown the presence of cognitive differences between men and women [33], particularly the phenomenon of hemispheric asymmetry during word processing between men and women: the left brain in men plays the major role, whereas both the left and right brains of women work simultaneously, which is exhibited through women's obvious advantages over men in speech generation [34]. In addition, women have better episodic memory and face recognition abilities than men [35]. Studies have shown that the working memory of patients with depression is impaired, which is manifested in the significant decrease in the accuracy of the working memory and the significantly prolonged reaction time of the working memory compared to the control group [36–39]. In this study, for both the accuracy (Figure 1) and the reaction time (Figure 2), there was no significant difference between the male and female patients. However, the accuracy in women was larger than that of men, and the reaction time in women was shorter than that of men. Although this difference was not statistically significant, it shows a trend: compared to men, women have more detailed emotional picture coding and a deeper level of emotional information processing; therefore, these emotional images had a greater effect on the cognition of women.

Studies on mood congruence suggest that information that is consistent with emotional stimuli is easier to remember [40] and that people in a negative emotional state are more inclined to remember negative information [41]. Therefore, compared to a healthy control group, patients with depression remember more negative stimuli data because these are consistent with their emotional state; likewise, they remember less positive stimuli data because these are inconsistent with their emotional state [42]. For both men and women in this study, the working memory for negative emotions was significantly stronger than that for positive emotions, and both genders showed memory effects of mood congruence. The study results illustrate that the memory effects of mood congruency are an important reason why patients with depression maintain their depression status.

Moreover, there are several potential reasons why we did not find any significant differences in terms of the emotional working memory of male and female patients: (1) the male and female patients in the study groups were from the outpatient department; they had depression of a lesser extent and were still able to work, live, and learn; (2) tasks of emotional working memory are relatively simple; and (3) the male and female groups were not large enough, which may have also affected the statistical validity.

Previous studies have shown that the brain's processing of external emotional information will lead to changes in pupil diameter. The pupil diameter dilated or constricted reflects people's emotional changes and emotional experiences [43–45]. This study used eye movement equipment to measure pupil diameter size when male and female patients with depression were viewing pictures of different emotions. Using pupil diameter for a neutral image as the baseline, we calculated the changes in pupil diameter when men and women were viewing pictures of positive and negative emotions.

The research results about pupil diameter changes showed that the main effects of gender were significant and women's emotional effects were significantly greater than those of men (Figure 3), indicating that women's emotional experiences for external emotional stimuli were far greater than those of men [46]. We further compared men's and women's pupil diameter changes when they were in different emotional tasks. We found that although the pupil diameter changes of the female group during positive emotions were greater than those of the male group, the difference was not significant, whereas the pupil diameter changes of the female group during negative emotions were significantly greater than those of the male group, indicating that women are more concerned about negative events than men. Depressed patients in emotional tasks have attentional bias [47] and processing bias [48] for negative information. Our results suggest that the negative emotional experiences of female patients with depression are significantly stronger than those of male patients, which increases the severity of depression in female patients. This is consistent with the result showing that the HAMD score of the female patients was larger than that of the male patients. The excessive negative emotional experiences of female patients with depression are possibly based on a physiological foundation: there is a greater synthesis of the emotion-related frontal cortex and 5-HT in certain subregions in the limbic system in women than in men [49]. One important feature of gender differences is different sex hormones: women primarily have estrogen and men primarily have androgen. Studies have shown that the high risk of female depression is related to the imbalance of female hormone such as ovarian hormone and progesterone, and the increase of estrogen has a direct relationship with negative emotions [3]. This occurs because, during negative stress, estrogen affects the balance of the hypothalamus-pituitary-HPA axis, leading to the female HPA axis imbalance and increased concentrations of the adrenal cortical hormone [50]. Meanwhile, excessive activity of the HPA axis easily arouses anxiety disorder and major depressive disorder [51–53]. However, there are studies suggesting that the gender difference is mainly due to the higher pressure to women during adolescence, premenstrual and perimenopausal periods, leading to the rise of depressive emotion [54, 55]. Recently, a perimenopausal depression model, proposed by Gordon et al. [1], has shown that the female depressive mood is caused by many factors, such as physiological factors (including estrogen fluctuations, GABA disorder, and HPA axis imbalance), female personality, and genetic vulnerability factors, as well as the psychological pressure; these physiological and social factors interacting with each other might lead to the occurrence of depression.

In addition, women's excessive emotional experiences to negative emotional stimuli can also come from the long-term accumulation of environmental pressure. There are gender differences in men's and women's abilities to respond to working conditions and the living environment and interpersonal skills. Hankin et al. [56] found that adolescent girls reported significantly more interpersonal and peer stressors than boys. Bouma et al. [57] found that girls were more likely to develop depressive symptoms in response to stress than were boys. These studies suggest that adolescent girls experience more objective and subjective psychological stressors than boys; particularly, interpersonal stress has been shown to partially mediate the increased prevalence of depression in girls after puberty [56, 58]. There are also gender differences in pressure venting. Women feel and accumulate more pressure to make themselves more susceptive to negative cognitive schemata, particularly in the face of negative events. Chronic stress increases anxiety and depression-like behavior [59–61]. Long-term stress can induce functional changes in the brain regions that involve anxiety and/or depression, including the parahippocampal gyrus, the amygdala, and the prefrontal cortex [30, 62]. Excessive pressure can cause anxiety-related symptoms [63] and the appearance and maintenance of severe depression [64]. Cognitive vulnerability transactional stress theory tends to support that depressive gender difference is due to sex cognitive vulnerability and stress [65]. The negative emotion caused by negative life events is often expanded by the female cognitive vulnerability. Meanwhile, facing the stress of negative events, men usually have more hostile and angry reaction, while women tend to meditate alone [66]. Meditation is not a proper way to deal with the pressure, because this way is more likely self-attribution, leading to depression [65]. Female cognitive vulnerability is increased by many factors such as female personality and genetic and estrogen factors. From adolescence especially, female hormone imbalance may result in the disorder of GABA and imbalance of HPA axis, combined with women's cognitive vulnerability, and improper ways to higher psychological pressure caused by the stress events, thus, further deepen negative cognitive process which may produce depressive mood disorder [1]. The present study has explained from the perspective of emotional cognition why females suffer from a longer term of depression and are more susceptive to relapse [6] and why women have a higher risk of severe depression episodes in the future [4, 5].

## 5. Conclusion

Our study results demonstrate no significant gender differences in emotional working memory among patients with depression of a lesser extent. However, both male and female patients have experienced memory effects of mood congruency, which was represented by the significantly higher accuracy of the negative emotion working memory than of the positive emotion working memory. In addition, gender differences were revealed in emotional experience as a response to external emotional stimuli. The level of emotional experience was higher in women than in men; in particular, women's negative emotional experiences were significantly stronger than those of men. The increased negative emotional experiences in women were likely an important reason why females suffer from longer term depression, are more susceptive to relapse, and have a higher risk of severe depression episodes.

## Figures and Tables

**Table 1:** Demographic data analysis.

	Male (mean ± SD)	Female (mean ± SD)	*p* value
Age (years)	34.63 ± 10.72	34.00 ± 10.19	0.90
Educational level (years)	13.63 ± 3.42	13.60 ± 3.78	0.99
HAMD (17 items)	19.63 ± 5.83	20.58 ± 6.14	0.01

SD: standard deviation; HAMD: Hamilton Depression Rating Scale.
